# Synthesis and Biological Evaluation of *N*-(5-(pyridin-2-yl)-1,3,4-thiadiazol-2-yl)benzamide Derivatives as Lipoxygenase Inhibitor with Potential Anticancer Activity

**Published:** 2017

**Authors:** Alireza Aliabadi, Ahmad Mohammadi-Farani, Sahar Roodabeh, Farahnaz Ahmadi

**Affiliations:** a *Pharmaceutical Sciences Research Center, School of Pharmacy, Kermanshah University of Medical Sciences, Kermanshah, Iran. *; b *Department of Medicinal Chemistry, Faculty of Pharmacy, Kermanshah University of Medical Sciences, Kermanshah, Iran. *; c *Department of Pharmacology, Toxicology and Medical Services, Faculty of Pharmacy, Kermanshah University of Medical Sciences, Kermanshah, Iran. *; d *Students Research Committee, Kermanshah University of Medical Sciences, Kermanshah, Iran.*

**Keywords:** Pyridine, 1, 3, 4-Thiadiazole, Synthesis, Lipoxygenase, MTT

## Abstract

In the recent years, the role of LOX enzymes in the origin of neoplastic diseases such as colorectal, skin, pancreatic and renal cancers has been confirmed. A new series of 1,3,4-thiadiazole derivatives bearing 2-pyridyl moiety was synthesized and the cytotoxicity of the members of this series was assessed using MTT protocol. Enzyme inhibitory activity of the prepared compounds was also tested against 15-lipoxygenase-1 as a novel target for the discovery of anticancer drugs. PC3, HT29 and SKNMC cell lines were utilized and the obtained results were compared with doxorubicin. Overall, nitro containing derivatives exerted a higher cytotoxic activity against PC3 cell line and methoxylated derivatives showed an acceptable activity against SKNMC cell line. Methoxylated derivatives were also the most potent enzyme inhibitors especially at position *ortho* of the phenyl residue.

## Introduction

Arachidonic acid (AA) and its metabolites have recently generated a heightened interest due to the growing evidence of their significant role in cancer biology (1). LOXs are a group of closely related non-heme iron containing dioxygenases. These enzymes catalyze the addition of molecular oxygen into poly-unsaturated fatty acids (PUFAs) containing cis, cis-1,4 pentadiene structures to give their hydroperoxy derivatives. LOXs are further classified into 5-, 8-, 9-, 11-, 12-, and 15-LOXs according to the positional specificity of arachidonate oxygenation (2-7). The LOXs convert polyunsaturated fatty acids like arachidonic and linoleic acids into biologically active metabolites that affect various cellular events such as signaling, structure and metabolism. According to the later tumorigenesis studies, it is likely that polyunsaturated fatty acids may enhance tumorigenesis via oxidative metabolism (8). Eicosanoids derived from the arachidonic acid cascade have been implicated in the pathogenesis of a variety of human diseases, including cancer, and are now believed to play important roles in tumor promotion, progression, and metastatic disease. These metabolites appear to alter cellular signaling pathways and thus the inappropriate expression might alter biological events and contribute to tumor development. The role of LOX enzymes in the cause of neoplastic diseases such as colorectal, skin, pancreatic and renal cancers has been confirmed (9, 10).There are two human forms of 15-LO, named 15-LO-1 and 15-LO-2. These enzymes have only 40% similarity in amino acid sequence and the biological function(s) of these enzymes is probably quite different. 15-LO-1 is highly transcriptionally regulated with narrow tissue distribution. The enzyme is predominantly expressed in airway epithelial cells, eosinophils, alveolar macrophages, dendritic cells and reticulocytes. The enzyme can catalyse the metabolism of arachidonic acid, and also certain other polyunsaturated fatty acids, to various metabolites. 15-LO-1 can also, in contrast to 5-LO and 12-LO, oxygenate fatty acids attached to membrane phospholipids. Interleukin (IL)-4 and IL-13 induce the expression of 15-LO-1 in cultivated monocytes, airway epithelial cells and mast cells, and in monocytes this induction could be inhibited by interferon- and glucocorticoids 

(11).Several compounds with 1,3,4-thiadiazole substructure exhibit pharmacological activities, including antimicrobial, anti-inflammatory, analgesic, antipyretic and especially anticancer activities. Therefore, interest in the syntheses of 1,3,4-thiadiazole derivatives as potential anticancer agents is significant in the current research of medicinal chemistry (12-16). According to our previous reports, the 1,3,4-thiadiazole derivatives act via several mechanisms for exertion of anticancer activity. Namely, inhibition of tyrosine kinase enzymes and induction of apoptosis are the main cellular mechanisms that have been observed for these compounds in our older research (17-19). In the current research, we focused on another probable mechanism for anticancer activity that has been reported recently for anticancer agents. In fact, the enzyme inhibitory potency of the synthesized compounds were investigated towards 15-lipoxygenase to reach novel anticancer lead compounds with capability the inhibition of 15-lipoxygense. 

## Experimental

*Chemistry*

**Scheme 1:**
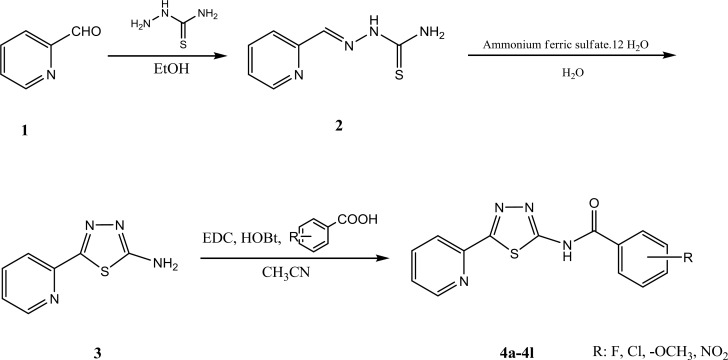
Synthetic protocol of compounds **4a-4l**

All chemical substances such as starting materials, reagents and solvents were purchased from commercial suppliers like Merck and Sigma-Aldrich companies. The purity of the prepared compounds was proved by thin layer chromatography (TLC) using various solvents of different polarities. Merck silica gel 60 GF_254_ plates were applied for analytical TLC. Column chromatography was performed on Merck silica gel (70-230 mesh) for purification of intermediate and final compounds. ^1^H-NMR spectra were recorded using a Varian 400 spectrometer, and chemical shifts are expressed as δ (ppm) with tetramethylsilane (TMS) as internal standard. The IR spectra were obtained on a Shimadzu 470 spectrophotometer (potassium bromide disks). Melting points were determined using elemental analyzer apparatus and are uncorrected. The mass spectra were run on a Finigan TSQ-70 spectrometer (Finigan, USA) at 70 eV. All cell lines were purchased from Pasteur Institute of Iran. All intermediate and final compounds were prepared according to the scheme 1. 

*Synthesis of 2-(pyridin-2-ylmethylene)hydrazinecarbothioamide (2)*

5 g (4.5 mL, 0.07 mmol) of pyridine-2-carbaldehyde were treated with 4.25 g (0.07 mmol) thiosemicarbazide in ethanol (100 mL) solvent. The reaction mixture was refluxed with addition of 1 mL of hydrochloric acid for 5 h. Thin layer chromatography (TLC) was utilized for monitoring the reaction progress. The formed precipitate was filtered and washed by cold water (20). 

^1^H NMR (DMSO-d_6_, 250 MHz) δ (ppm): 7.42 (t, 1H, H_5_-Pyridine), 7.93 (t, 1H, H_4_-Pyridine), 8.02 (s, 1H, Pyridine-CH=N-), 8.29 (d, 1H, H_3_-Pyridine), 8.41 (brs, NH_2_), 8.76 (d, 1H, H6-Pyridine), 11.73 (brs, NH). IR (KBr, cm^-1^) ῡ: 3433 (NH, Stretch), 3259, 3159 (NH_2_, Stretch), 3055 (C-H, Aromatic, Stretch), 1608 (C=C, Aromatic, Stretch), 1527 (N-H, Bend), 1462 (C=C, Aromatic, Stretch), 1296 (C-N, Stretch). MS (*m/z*, %): 180 (M^+^, 100), 120 (90), 102 (15), 105 (20), 92 (75), 78 (40), 65 (85). 

*Synthesis of 5-(pyridin-2-yl)-1,3,4-thiadiazol-2-amine *(3)

5 g (0.027 mmol) of 2-(pyridin-2-ylmethylene)hydrazinecarbothioamide (2) was refluxed for 1 h with 5 g ammonium ferric sulfate [(NH_4_Fe(SO_4_)_2_.12 H_2_O] in water (100 mL) as solvent. Then, 10 g of ammonium ferric sulfate [(NH_4_Fe(SO_4_)_2_.12 H_2_O] was dissolved in 30 mL of water and added to the reaction medium. The reflux condition was continued for 30 h. Thin layer chromatography (TLC) was applied for monitoring the reaction. The reaction mixture was poured in separating funnel and ethyl acetate was added. Organic phase was washed three times by sodium bicarbonate 2% and brine. Dryness was carried out over anhydrous sodium sulfate and then filtered. The ethyl acetate was evaporated under reduced pressure and obtained powder was washed by *n*-hexane and diethyl ether (20).

^1^H NMR (DMSO-d_6_, 250 MHz) δ (ppm): 7.40 (t, 1H, H_5_-Pyridine), 7.51 (s, 2H, NH_2_), 7.89 (t, 1H, H_4_-Pyridine), 8.03 (d, 1H, H_3_-Pyridine), 8.56 (d, 1H, H_6_-Pyridine). IR (KBr, cm^-1^) ῡ: 3271 (NH_2_, Stretch), 3093 (C-H, Aromatic, Stretch), 1620 (C=C, Aromatic, Stretch), 1500 (N-H, Bend), 1435 (C=C, Aromatic, Stretch), 1130 (C-N, Stretch). MS (*m/z*, %): 178 (M^+^, 100), 136 (15), 120 (20), 105 (40), 92 (20), 78 (60). 

*General procedure for synthesis of compounds *4a-4l:

In a flat bottom flask, 1.1 mmol of appropriate benzoic acid derivative was reacted with 152 mg (1.1 mmol) hydroxybenzotriazole (HOBt) and *N*-ethyl-*N*-dimethylaminopropyl carbodiimide (EDC) in acetonitrile (20 mL). The reaction mixture was stirred at room temperature for 30 min. Then, 200 mg (1.1 mmol) of compound (3) was added to the reaction medium and stirring was continued for 24 h. Acetonitrile was evaporated under reduced pressure and ethylacetate/water (25/25 mL) was added to the residue. Organic layer was washed two times by sodium bicarbonate (2%) and brine. Anhydrous sodium sulfate was used for dryness and then filtered. The ethyl acetate was evaporated using rotary evaporator apparatus and the obtained powder was washed by *n*-hexane and diethyl ether (Et_2_O) (17-19, 21). 

*2-Chloro-N-(5-(pyridin-2-yl)-1,3,4-thiadiazol-2-yl)benzamide *(4a)

^1^H NMR (DMSO-d_6_, 250 MHz) δ (ppm): 7.58 (m, 4H, Aromatic), 7.70 (d, 1H, *J *= 7.5 Hz, H_3_-2-Chlorophenyl), 8.00 (t, 1H, H_4_-Pyridine), 8.23 (d, 1H, *J* = 7.5 Hz, H_3_-Pyridine), 8.69 (d, 1H, H6-Pyridine), 13.33 (brs, NH). IR (KBr, cm^-1^) ῡ: 3429 (N-H, Stretch), 3116 (C-H, Aromatic), 1678 (C=O, Stretch), 1585 (C=C, Aromatic, Stretch), 1535 (N-H, Bend), 1435 (C=C, Aromatic, Stretch), 1311 (C-N, Stretch). MS (*m/z*, %): 318 (M^+^+2, 5), 316 (M^+^, 2), 281 (85), 253 (20), 141 (55), 139 (100), 122 (20), 111 (60), 78 (40), 51 (20).

**Table 1:** Properties of synthesized compounds

Compound	R	Closed fromula	MW (g/mol)	mp (°C)	Yield (%)
**4a**	2-Cl	C_14_H_9_ClN_4_OS	316.77	250	37
**4b**	3-Cl	C_14_H_9_ClN_4_OS	316.77	286-289	56
**4c**	4-Cl	C_14_H_9_ClN_4_OS	316.77	366	75
**4d**	2-F	C_14_H_9_FN_4_OS	300.31	242	55
**4e**	3-F	C_14_H_9_FN_4_OS	300.31	321	53
**4f**	4-F	C_14_H_9_FN_4_OS	300.31	321	43
**4g**	2-NO_2_	C_14_H_9_N_5_O_3_S	327.32	342	51
**4h**	3-NO_2_	C_14_H_9_N_5_O_3_S	327.32	302	67
**4i**	4-NO_2_	C_14_H_9_N_5_O_3_S	327.32	390	74
**4j**	2-OCH_3_	C_15_H_12_N_4_O_2_S	312.35	228	19
**4k**	3-OCH_3_	C_15_H_12_N_4_O_2_S	312.35	250	34
**4l**	4-OCH_3_	C_15_H_12_N_4_O_2_S	312.35	210	75

**Table 2:** Cytotoxicity and enzymatic results of compounds **4a-4l**. Cytotoxicty results were presented as IC_50_ (µM) and enzymatic results were provided as percent of inhibition at 200 (µM) concentration

R	Compounds	PC3	HT29	SKNMC	15-Lipoxygenase-1
4a	2-Cl	79.24	49.50	22.41	8
4b	3-Cl	17.71	45.66	29.78	8
4c	4-Cl	49.08	66.94	6.71	12
4d	2-F	76.78	65.58	99.64	17
4e	3-F	74.58	73.77	70.50	13
4f	4-F	28.66	58.42	64.33	25
4g	2-NO_2_	35.30	6.52	54.44	5
4h	3-NO_2_	59.26	3.28	49.21	5
4i	4-NO_2_	37.49	4.01	56.40	ND*
4j	2-OCH_3_	4.96	16.00	15.28	28
4k	3-OCH_3_	40.70	73.02	31.89	26
4l	4-OCH_3_	9.81	24.20	31.16	14
Doxorubicin	-	3.8	2.1	1.3	
Quercetin	-	-	-	-	100

* ND: Not dissolved.

*3-Chloro-N-(5-(pyridin-2-yl)-1,3,4-thiadiazol-2-yl)benzamide* (4b)

^1^H NMR (DMSO-d_6_, 250 MHz) δ (ppm): 7.53 (t, 1H, H_5_-3-Chlorophenyl), 7.61 (d, 1H, *J *= 7.5 Hz, H_6_-3-Chlorophenyl), 7.72 (t, 1H, H_5_-Pyridine), 7.99 (t, 1H, H_4_-Pyridine), 8.08 (d, 1H, *J* = 7.5 Hz, H_3_-Pyridine), 8.20 (s, 1H, H_2_-3-Chlorophenyl), 8.23 (d, 1H, *J* = 7.5 Hz, H_4_-3-Chlorophenyl), 8.69 (d, 1H, *J* = 7.5 Hz, H_6_-Pyridine), 12.50 (brs, NH). IR (KBr, cm^-1^) ῡ: 3147 (N-H, Stretch), 3005 (C-H, Aromatic, Stretch), 1674 (C=O, Stretch), 1535 (N-H, Bend), 1315 (C-N, Stretch). MS (*m/z*, %): 318 (M^+^+2, 10), 316 (M^+^, 4), 315 (15), 288 (20), 211 (20), 141 (25), 139 (100), 122 (25), 111 (75), 95 (10), 78 (50), 75 (25), 51 (20).

*4-Chloro-N-(5-(pyridin-2-yl)-1,3,4-thiadiazol-2-yl)benzamide* (4c)

^1^H NMR (DMSO-d_6_, 250 MHz) δ (ppm): 7.38 (t, 1H, H_5_-Pyridine), 7.47 (d, 2H, *J* = 10 Hz, H_2,6_-4-Chlorophenyl), 7.89 (t, 1H, H_4_-Pyridine), 8.16 (d, 3H, H_3_-Pyridine, H_3,5_-4-Chlorophenyl), 8.60 (d, 1H, H_6_-Pyridine). IR (KBr, cm^-1^) ῡ: 3159 (N-H, Stretch), 3089, 3062, 3008 (C-H, Aromatic, Stretch), 1670 (C=O, Stretch), 1589 (C=C, Aromatic, Stretch), 1523 (N-H, Bend), 1485 (C=C, Aromatic, Stretch), 1311 (C-N, Stretch). MS (*m/z*, %): 318 (M^+^+2, 10), 316 (M^+^), 315 (30), 288 (60), 149 (60), 139 (100), 111 (40), 78 (20), 57 (20). 

*2-Fluoro-N-(5-(pyridin-2-yl)-1,3,4-thiadiazol-2-yl)benzamide* (4d)

^1^H NMR (DMSO-d_6_, 250 MHz) δ (ppm): 7.37 (m, 2H, H_5_, H_4_-Pyridine), 7.54 (t, 1H, H_6_-2-Fluorophenyl), 7.63 (t, 1H, H_4_-2-Fluorophenyl), 7.79 (t, 1H, H_5_-2-Fluorophenyl), 8.00 (t, 1H, H_3_-2-Fluorophenyl), 13.17 (brs, NH). IR (KBr, cm^-1^) ῡ: 3120 (N-H, Stretch), 3070 (C-H, Aromatic, Stretch), 1685 (C=O, Stretch), 1616 (C=C, Aromatic, Stretch), 1535 (N-H, Bend), 1496 (C=C, Aromatic, Stretch), 1319 (C-N, Stretch). MS (*m/z*, %): 300 (40, M^+^), 281 (75), 272 (40), 253 (15), 123 (100), 95 (75), 78 (30), 76 (25), 51 (15).

*3-Fluoro-N-(5-(pyridin-2-yl)-1,3,4-thiadiazol-2-yl)benzamide* (4e)

^1^H NMR (DMSO-d_6_, 250 MHz) δ (ppm): 7.56 (m, 3H, Aromatic), 8.00 (m, 3H, Aromatic), 8.24 (d, 1H, H_3_-Pyridine), 8.69 (d, 1H, H_6_-Pyridine), 13.31 (brs, NH). IR (KBr, cm^-1^) ῡ: 3132 (N-H, Stretch), 3070 (C-H, Aromatic, Stretch), 1678 (C=O, Stretch) 1589 (C=C, Aromatic, Stretch), 1531 (N-H, Bend), 1489 (C=C, Aromatic, Stretch), 1269 (C-N, Stretch). MS (*m/z*, %): 300 (15, M^+^), 299 (20), 272 (55), 123 (100), 95 (90), 78 (40), 51 (20).

*4-Fluoro-N-(5-(pyridin-2-yl)-1,3,4-thiadiazol-2-yl)benzamide* (4f)

^1^H NMR (DMSO-d_6_, 250 MHz) δ (ppm): 7.39 (t, 2H, H_2,6_-4-Fluorophenyl), 7.53 (t, 1H, H_5_-Pyridine), 7.99 (t, 1H, H_4_-Pyridine), 8.23 (3H, Aromatic), 8.68 (d, H_6_-Pyridine), 13.35 (brs, NH). IR (KBr, cm^-1^) ῡ: 3151 (N-H, Stretch), 3066 (C-H, Aromatic, Stretch), 1678 (C=O, Stretch), 1604 (C=C, Aromatic, Stretch), 1539 (N-H, Bend), 1234 (C-N, Stretch). MS (*m/z*, %): 300 (20, M^+^), 299 (30), 272 (40), 123 (100), 95 (40), 78 (20). 

*2-Methoxy-N-(5-(pyridin-2-yl)-1,3,4-thiadiazol-2-yl)benzamide* (4g)

^1^H NMR (DMSO-d_6_, 250 MHz) δ (ppm): 3.91 (s, 3H, -OCH_3_), 7.09 (t, 1H, H_5_-2-Methoxyphenyl), 7.22 (d, 1H, H_3_-2-Methoxyphenyl), 7.56 (m, 2H, H_5_-Pyridine, H_4_-2-Methoxyphenyl), 7.70 (d, 2H, H_6_-2-Methoxyphenyl), 7.99 (t, 1H, H_4_-Pyridine), 8.23 (d, 1H, H_3_-Pyridine), 8.69 (d, 1H, H_6_-Pyridine), 12.44 (brs, NH). IR (KBr, cm^-1^) ῡ: 3294 (N-H, Stretch), 3070 (C-H, Aromatic, Stretch), 1666 (C=O, Stretch), 1600 (C=C, Aromatic, Stretch), 1523 (N-H, Bend), 1485 (C=C, Aromatic, Stretch), 1238 (C-N, Stretch). MS (*m/z*, %): 312 (M^+^, 10), 282 (30), 281 (100), 136 (20), 135 (100), 92 (35), 78 (35), 77 (75), 51 (15).

*3-Methoxy-N-(5-(pyridin-2-yl)-1,3,4-thiadiazol-2-yl)benzamide* (4h)

^1^H NMR (DMSO-d_6_, 250 MHz) δ (ppm): 3.85 (s, 3H, -OCH_3_), 7.22 (d, 2H, *J* = 7.5 Hz, H_6_-3-Methoxyphenyl), 7.43 (t, 1H, H_5_-3-Methoxyphenyl), 7.53 (d, 2H, *J* = 7.5 Hz, H_6_-3-Methoxyphenyl), 7.71 (s, H_2_-3-Methoxyphenyl), 7.73 (t, 1H, H_5_-Pyridine), 7.99 (t, 1H, H_4_-Pyridine), 8.23 (d, 1H, H_3_-Pyridine), 8.69 (d, 1H, H6-Pyridine), 13.20 (brs, NH). IR (KBr, cm^-1^) ῡ: 3109 (NH, Stretch), 3051 (C-H, Aromatic, Stretch), 2920, 2846 (C-H, Aliphatic, Stretch), 1670 (C=O, Stretch), 1585 (C=C, Aromatic, Stretch), 1531 (N-H, Bend), 1492 (C=C, Aromatic, Stretch), 1276 (C-N, Stretch). MS (*m/z*, %): 312 (M^+^, 15), 284 (25), 135 (100), 122 (15), 107 (5), 92 (35), 77 (20). 

*4-Methoxy-N-(5-(pyridin-2-yl)-1,3,4-thiadiazol-2-yl)benzamide* (4i)

^1^H NMR (DMSO-d_6_, 250 MHz) δ (ppm): 3.81 (s, 3H, -OCH_3_), 6.99 (d, 2H, *J* = 7.5 Hz, H_3,5_-4-Methoxyphenyl), 7.36 (t, 1H, H_5_-Pyridine), 7.51 (m, 1H, Pyridine), 7.69 (d, 2H, *J* = 7.5 Hz, H_2,6_-4-Methoxyphenyl), 8.05 (m, 3H, Pyridine), 8.56 (d, 1H, H6-Pyridine), 13.25 (brs, NH). IR (KBr, cm^-1^) ῡ: 3275 (N-H, Stretch), 3097, 3066 (C-H, Aromatic, Stretch), 2931 (C-H, Aliphatic, Stretch), 1662 (C=O, Stretch), 1604 (C=C, Aromatic, Stretch), 1261 (C-N, Stretch). 

MS (*m/z*, %): 312 (M^+^, 10), 284 (10), 135 (100), 122 (10), 107 (15), 92 (15), 77 (20). 

*2-Nitro-N-(5-(pyridin-2-yl)-1,3,4-thiadiazol-2-yl)benzamide *(4j)

^1^H NMR (DMSO-d_6_, 250 MHz) δ (ppm): 7.55 (t, 1H, H_5_-Pyridine), 7.82-7.92 (m, 3H, Aromatic), 8.02 (t, 1H, H_3_-Pyridine), 8.23 (t, 2H, Aromatic), 8.69 (d, 1H, H_6_-Pyridine), 13.45 (brs, NH). IR (KBr, cm^-1^) ῡ: 3097 (C-H, Aromatic, Stretch), 2997, 2897 (C-H, Aliphatic, Stretch), 1678 (C=O, Stretch), 1531 (NO_2_, Stretch, Asymmetric), 1311 (NO_2_, Stretch, 

Symmetric). MS (*m/z*, %): 327 (12, M^+^), 299 (20), 281 (30), 253 (25), 205 (15), 150 (75), 122 (100), 104 (20), 78 (75), 76 (40), 51 (45).

*3-Nitro-N-(5-(pyridin-2-yl)-1,3,4-thiadiazol-2-yl)benzamide* (4k)

^1^H NMR (DMSO-d_6_, 250 MHz) δ (ppm): 7.54 (t, 1H, H_5_-Pyridine), 7.86 (t, 1H, *J* = 7.5 Hz, H_5_-3-Nitrophenyl), 8.02 (t, 1H, H_4_-Pyridine), 8.23 (d, 1H, H_4_-3-Nitrophenyl), 8.47 (d, 1H, H_3_-Pyridine), 8.53 (d, 1H, H_6_-3-Nitrophenyl), 8.69 (d, 1H, H_6_-Pyridine), 8.98 (s, 1H, H_2_-3-Nitrophenyl), 13.50 (brs, NH). IR (KBr, cm^-1^) ῡ: 3093 (C-H, Aromatic, Stretch), 2997, 2920 (C-H, Aromatic, Stretch), 1674 (C=O, Stretch), 1531 (NO_2_, Asymmetric, Stretch), 1323 (NO_2_, Symmetric, Stretch). MS (*m/z*, %): 327 (25, M^+^), 326 (45), 299 (50), 240 (15), 205 (40), 150 (100), 122 (35), 104 (50), 78 (45), 76 (50), 50 (15).

*4-Nitro-N-(5-(pyridin-2-yl)-1,3,4-thiadiazol-2-yl)benzamide* (4l)

^1^H NMR (DMSO-d_6_, 250 MHz) δ (ppm): 7.51 (t, 1H, H_5_-Pyridine), 7.68 (dd, 4H, 4-Nitrophenyl), 7.98 (t, 1H, H_4_-Pyridine), 8.21 (d, 1H, H_3_-Pyridine), 8.67 (d, 1H, H6-Pyridine). IR (KBr, cm^-1^) ῡ: 3136 (NH, Stretch), 3070 (C-H, Aromatic, Stretch), 2924 (C-H, Aliphatic, Stretch), 1685 (C=O, Stretch), 1527 (NO_2_, Asymmetric, Stretch), 1319 (NO_2_, Symmetric, Stretch). MS (*m/z*, %): 327 (20, M^+^), 299 (30), 240 (80), 205 (20), 178 (60), 167 (50), 149 (90), 136 (25), 123 (60), 109 (40), 97 (60), 83 (60), 69 (75), 57 (100). 

*MTT assay*

Synthesized derivatives of 1,3,4-thiadiazole (compounds 4a-4l) were tested for cytotoxic activity at 0.1-250 mcg/mL concentration in three human cancer cell lines of PC3 cell (prostate cancer), HT-29 (Colon cancer) and SKNMC (Neuroblastoma). Cells from different cell lines were seeded in 96-well plates at the density of 8000–10,000 viable cells per well and incubated for 48 h to allow cell attachment. The cells were then incubated for another 48-96 h (depends to cell cycle of each cell line) with various concentrations of compounds 4a-4l. Cells were then washed in PBS, and 20 μL of MTT (3-(4, 5-dimethylthiazol-2-yl)-2,5-diphenyl tetrazolium bromide solution (5 mg/mL) were added to each well. An additional 4 h of incubation at 37 ^o^C were done, and then the medium was discarded. Dimethyl sulfoxide (60 μL) was added to each well, and the solution was vigorously mixed to dissolve the purple tetrazolium crystals. The absorbance of each well was measured by plate reader (Anthous 2020; Austria) at a test wavelength of 550 nm against a standard reference solution at 690 nm. The amount of produced purple formazan is proportional to the number of viable cells 

(17-19).

*Enzymatic assay*

The basis of this method is oxidative coupling of 3-methyl-2-benzothiazolinone hydrazone (MBTH) with 3-(dimethylamino) benzoic acid (DMAB) in a hemoglobin catalyzed reaction. This reaction is initiated in the presence of lipoxygenase reaction product, linoleic acid hydroperoxide and results in a blue color formation which has a peak absorbtion at 590 nm (22). Quercetin was used as the reference compound. Linoleic acid and two stock solutions (A and B) were prepared first. Solution A contained 50 mM DMAB and l00 mM phosphate buffer (pH = 7.0). Solution B was prepared by mixing 10 mM MBTH (3 mL) and hemoglobin (5 mg/mL, 3 mL) in 50 mM phosphate buffer at pH 5.0 (25 mL). A linoleic acid solution (1mg/mL) was prepared by diluting 5 mg linoleic acid (solubilised in 0.5 mL ethanol) with KOH 100 mM. For each compound the samples were solved in ethanol (25 µL) and mixed in a test tube with SLO (4000 units/mL, prepared in 50 mM phosphate buffer pH = 7.0, 25 µL) and phosphate buffer (50 mM, pH = 7, 900 µL). After a 5 min delay at room temperature, 50 µL linoleic acid was added to the mixture to start the hydroperoxidation reaction. After 8 min, solution A (270 µL) and solution B (130 µL) were added to the above mixture. 5 min later, 200 µL of SDS solution (2%) was added to stop the reaction. The absorbance at 590 nm was compared with control (ethanol without sample).

## Results and Discussion

*Cytotoxicity assay*

According to the Table 2. all prepared derivatives were tested against three cancerous cell lines. PC3 (prostate carcinoma), HT29 (colorectal cancer) and SKNMC (neuroblastoma) were utilized to assess the potentiality of cytotoxicity *in-vitro*. Tested compounds rendered a high anticancer activity towards HT29 cell line compared to other cell lines. Nitro containing derivatives (4g, 4h, 4i) demonstrated a remarkable inhibitory activity against HT29 cell line. *Meta* positioning of the nitro moiety caused a better cytotoxic potency than other positions. It is likely that the capability of the nitro group for generating free radicals is responsible for its higher anticancer potency in comparison with other moieties. Generally, investigation of the various substituents with electron withdrawing (F, Cl, NO_2_) as well as electron donating (-OCH_3_) activity were carried out. Chlorinated derivatives showed superior activity against SKNMC cell line than PC3 and HT29. Probably, enhancement of the lipophilic property of these compounds that caused by chlorine substituent is a favorable parameter for improving the anticancer activity. *Para* positioning of the chlorine atom led to the significant cytotoxic activity towards SKNMC cell line (IC_50_ = 6.71). A significant potency was observed against PC3 cell line while methoxylated derivatives were investigated. The methoxy moiety showed a superior activity when introduced on the *ortho* and *para* positions of the phenyl residue. It is clear that electron donating feature of this group is a beneficial factor for increasing the activity. Movement of the methoxy to the *meta* position of the phenyl residue end to an outstanding reduction in potency. 

*Enzymatic assay*

Inhibitory activity of synthesized derivatives 4a-4l was examined against 15-LOX-1 enzyme and obtained results were compared to quercetin as reference compound. Compounds 4j (*o*-methoxy) and 4k (*m*-methoxy) as methoxylated derivatives displayed the best inhibitory activity in this series. Nitro containing derivatives showed the lowest enzyme inhibitory activity. Besides, fluorinated and chlorinated derivatives were also tested to explore the role of electron withdrawing effect of the moiety. Position *para* was the best position for fluorine atom as well as chlorine atom to inhibit the lipoxygenase enzyme. Fluorine demonstrated a higher inhibitory potency at position *para* than chlorine moiety. It is probable increasing the electron withdrawing effect of the moiety and also reducing the lipophilicity are the responsible factors for this evidence.

*The authors have been declared no conflict of interest.*
